# Targeted Genomic Region Masking Supports Accurate Variant Calling While Suppressing Low-Complexity Sequencing Artifacts

**DOI:** 10.3390/genes17070772

**Published:** 2026-06-30

**Authors:** Chrysoula Kaligerou, Athina Tsagkalidou, Vasiliki Pogka, Dimitrios Christos Tremoulis, Timokratis Karamitros

**Affiliations:** Bioinformatics and Applied Genomics Unit, Department of Microbiology, Hellenic Pasteur Institute, 11521 Athens, Greece; c.kaligerou@pasteur.gr (C.K.); a.tsagkalidou@pasteur.gr (A.T.); vpoga@pasteur.gr (V.P.); dimtremoulis@pasteur.gr (D.C.T.)

**Keywords:** variant calling, clinical genomics, low-complexity regions, spatial masking, analytical reference standards, sequencing artifacts, dbSNP paradox

## Abstract

**Background:** False-positive variant calls generated within low-complexity regions (LCRs) remain a persistent bottleneck in clinical genomics, complicating downstream analysis. This study evaluates a targeted spatial masking strategy designed to suppress deterministic artifacts in short-read sequencing data, while preserving clinically actionable variants residing outside LCRs. We implemented a selective masking protocol prior to variant calling across analytical reference standards (EQA, NA12878) and two independent breast cancer whole-exome sequencing cohorts (*n* = 25). **Methods:** Callsets were evaluated for diagnostic sensitivity, precision gains, mutational signatures, VAF behavior, pseudo-multiallelic noise and ClinVar/dbSNP annotation. **Results:** The protocol removed thousands of sequencing and alignment artifacts while maintaining the retained biological callset, with negligible disease-associated diagnostic variants detected in the excluded artifact fraction. LCR masking preserved physiological Ti/Tv and Ins/Del profiles in retained calls, resolved pseudo-multiallelic noise, and distinguished excluded artifact calls by distorted mutational and VAF signatures. dbSNP profiling showed cohort-dependent behavior: TCGA-BRCA reproduced an intriguing phenomenon, with excluded calls showing higher dbSNP annotation than retained calls, whereas AURORA showed the opposite direction. **Conclusions:** These findings demonstrate the potential vulnerability of one-dimensional database annotation for variant authentication and highlight targeted spatial filtration as a critical, early pipeline intervention for high-fidelity clinical genomics of non-LCR-associated germline variants using short reads.

## 1. Introduction

Next-Generation Sequencing (NGS) has significantly changed clinical diagnostics, enabling the rapid characterization of genomic variation across diverse disease phenotypes [1,2]. Even though algorithms have improved, Low-Complexity Regions (LCRs), such as simple tandem repeats, and homopolymer tracts still act as a bottleneck to accurately identify genetic variation [3,4]. These regions naturally disrupt library preparation kinetics and create severe mapping ambiguities during downstream bioinformatic alignment, regardless of the underlying enrichment chemistry, which leads to a significant number of False-Positive (FP) variant calls [5].

Crucially, this technical vulnerability is frequently embedded at the assay design level. In targeted enrichment strategies, such as hybrid-capture Whole-Exome Sequencing (WES) or clinical amplicon panels, probe synthesis relies on standardized algorithmic and thermodynamic rules to maximize target affinity and sequencing depth [6,7]. Due to the computational and logistical simplicity of designing continuous tiling arrays based on these generic parameters, probe construction rarely considers or excludes LCRs. Therefore, standard diagnostic assays unintentionally capture and sequence these repetitive elements, integrating the noise into the raw sequencing data and shifting the burden of artifact resolution entirely onto downstream bioinformatics pipelines [8].

To trace the path of this noise, clinical workflows routinely rely on foundational annotation databases, such as dbSNP and large-scale population aggregates [9,10]. They use cataloged reference (rs) IDs and allele frequencies as proxies for variant authenticity. However, this paradigm harbors a critical blind spot: because LCR-induced artifacts are driven by the underlying genomic architecture rather than stochastic sequencing errors, they are highly reproducible [11]. Consequently, reproducible mapping inaccuracies arise consistently across global genomic studies [12] and are archived in public repositories, establishing a feedback loop that amplifies the prevalence of false-positive variants in centralized datasets.

In the present study, we systematically profile the structural and clinical signatures of LCR-induced technical noise within the inherent constraints of standard short-read sequencing technologies. Utilizing analytical reference standards alongside WES cohorts, we demonstrate that implementing a targeted spatial masking strategy safely suppresses deterministic artifacts and restores physiological mutational signatures. Consequently, we explicitly frame this methodology around the optimal detection of germline variants (SNPs and INDELs) associated with known clinical diseases that reside outside of LCRs. By eliminating the artifactual noise floor and resolving ambiguous multi-allelic clutter, this masking protocol allows true-positive signals to easily meet mapping quality thresholds. Ultimately, while we acknowledge that resolving potentially pathogenic variants entirely embedded within LCRs remains a fundamental limitation of short-read lengths, our approach highlights spatial filtration as a critical, necessary component for high-fidelity clinical genomics in all other regions.

## 2. Materials and Methods

The experimental design of this study was structured to systematically evaluate the technical and clinical impact of targeted spatial masking within highly repetitive genomic intervals. Our methodology was divided into two distinct phases: (1) analytical validation utilizing established reference standards—a practice recognized as the optimal gold standard for benchmarking variant caller performance [13]—to precisely quantify diagnostic sensitivity and precision, and (2) clinical cohort profiling to assess the real-world restoration of mutational signatures and the control of database-annotated noise. The following subsections detail the sequencing strategies, computational filtration protocols, and statistical frameworks employed to execute this dual-phase validation.

### 2.1. Cohort Selection and Sequencing Strategies

To thoroughly evaluate the spatial masking strategy across varying assay architectures, we utilized analytical reference standards alongside a clinical cohort. Analytical validation was performed using the Genome in a Bottle (GIAB) NA12878 reference standard (Coriell Institute for Medical Research, Camden, NJ, USA) [3] and an External Quality Assessment (EQA) DNA SEQUENCING-NGS (Germline SNVs and INDELs) reference standard (European Molecular Genetics Quality Network [EMQN], Manchester, UK). To assess technical reproducibility, these samples were sequenced in-house with controlled replication (NA12878 in triplicate; EQA in duplicate) on the Illumina NextSeq 2000 platform (Illumina, Inc., San Diego, CA, USA). Two distinct library preparation strategies were evaluated:

1. Hybridization-based Whole-Exome Sequencing (WES): Libraries were prepared using the Illumina DNA Prep with Enrichment kit (Illumina, Inc., San Diego, CA, USA) combined with the Twist Exome 2.5 Panel (Twist Bioscience, South San Francisco, CA, USA), targeting ∼37.5 Mb of coding regions. Sequencing was performed using paired-end 2 × 200 bp reads to achieve a mean on-target coverage of ∼180–230×.
2. Amplicon-based Targeted Panel: Libraries were prepared using the QIAseq Targeted DNA Pro Panel (PHS-001Z; QIAGEN, Hilden, Germany). Sequencing was performed using paired-end 2 × 150 bp reads, achieving high-depth coverage (>350×) across target regions.

To evaluate the scalability of the protocol on real-world datasets, a clinical cohort including 10 normal blood samples was retrieved from the TCGA-BRCA whole-exome sequencing dataset from the database of Genotypes and Phenotypes (dbGaP) under accession number phs000178.v11.p8 (https://www.ncbi.nlm.nih.gov/projects/gap/cgi-bin/study.cgi?study_id=phs000178.v11.p8 accessed on 14 November 2025). In addition, 15 normal blood WES clinical samples were retrieved from the AURORA US Metastatic Breast Cancer Retrospective Project, obtained from dbGaP under accession number phs002622.v1.p1, (https://www.ncbi.nlm.nih.gov/projects/gap/cgi-bin/study.cgi?study_id=phs002622.v1.p1, accessed on 14 November 2025). Computational analysis of in-house sequenced targeted panel data was performed using the GRCh38 reference assembly, while in-house sequenced hybrid-capture exome data was analyzed using GRCh37 assembly (Table 1). TCGA-BRCA and AURORA US WES data were analyzed using the GRCh38 assembly (Table 2 and Table 3).

### 2.2. Upstream Spatial Masking Strategy

A critical methodological distinction of our approach is the stage of intervention and the precision of the exclusion targets. Conventional masking and filtering strategies, such as the application of ENCODE Blacklists [14] or Genome in a Bottle (GIAB) difficult-region stratifications [15], are highly effective but are typically implemented purely downstream by intersecting generated VCF files with exclusion BED files. Similarly, post-calling statistical modeling (e.g., Variant Quality Score Recalibration, VQSR) [16] evaluates variants only after they have been processed by the caller. In our pipeline, we do not replace these essential downstream tools; rather, we bulletproof the methodology by introducing a synergistic, strictly upstream spatial masking step (pre-variant calling). The spatial coordinates of the defined Low-Complexity Regions (LCRs) were compiled into exclusion BED files and supplied directly to the variant calling algorithms. By proactively restricting the caller’s search space, this design prevents the computational processing of severely ambiguous alignments. Consequently, this upstream exclusion protects downstream statistical models from being overwhelmed by deterministic noise, significantly reducing the generation of false-positive single nucleotide variants (SNVs), artifactual insertions and deletions (INDELs), and complex pseudo-multiallelic clutter.

### 2.3. Creation of a Curated Set of Genomic Coordinates and Filtration

#### 2.3.1. Identification and Retrieval of Repetitive Genomic Elements

To evaluate the impact of low-complexity region (LCR) masking comprehensively across diverse sequencing strategies and large-scale cohorts, a standardized germline variant calling pipeline was deployed. The analysis encompassed both WES and targeted panel workflows. Crucially, while comprehensive resources like full GIAB stratifications [17] or complete RepeatMasker [18] tracks provide invaluable data for global benchmarking, deploying these broad BED files directly as an upstream clinical mask is impractical. Their broad scope encompasses complex repeats, transposable elements, and segmental duplications, which would result in an excessively conservative filter, inadvertently masking numerous clinically relevant variants. Therefore, our spatial masking was strictly targeted on Homopolymer tracts and Simple Repeats.

Homopolymer coordinates and length thresholds were obtained from the Genome in a Bottle (GIAB) v3.0 Low-Complexity Stratifications resource [19]. For the filtration of homopolymer-induced noise, specialized stratification files for both GRCh37 and GRCh38 assemblies were retrieved from the GIAB v3.0 genomic stratifications repository (https://ftp-trace.ncbi.nlm.nih.gov/ReferenceSamples/giab/release/genome-stratifications/v3.0/, accessed on 28 September 2025). Specifically, the selected intervals include exclusively homopolymers greater than 6 bp in length and imperfect homopolymers greater than 10 bp. To identify repetitive genomic elements, the RepeatMasker (rmsk) tables for the GRCh38 (hg38) and GRCh37 (hg19) assemblies were retrieved from the UCSC GoldenPath database (https://hgdownload.soe.ucsc.edu/downloads.html, accessed on 28 September 2025) [18]. Simple, low-entropy motifs inherently prone to alignment ambiguity under the Simple Repeats class (e.g., micro-satellites and short tandem repeats-STRs) were masked, in order to establish the curated set of exclusion intervals. Complex repetitive elements with high informational entropy (e.g., Alu and LINE elements) were retained, as their internal sequence diversity provides unique anchoring for high-confidence read alignment.

#### 2.3.2. Elimination of Edge-Effect Artifacts

To prevent edge-effect artifacts during local de novo assembly, a 1-bp spatial buffer was applied to both simple motif and homopolymer coordinates using bedtools slop (v2.31.1). Finally, these buffered simple motifs and homopolymer coordinates were removed using bedtools from the primary regions of interest (ROIs). This sequential process ensured that the final BED files contain only high-complexity genomic intervals, minimizing the risk of stutter-induced artifacts during local de novo assembly.

### 2.4. Orthogonal Validation Using Analytical Reference Standards

To independently validate that the variants excluded by our spatial masking strategy were indeed technical artifacts rather than genuine biological variations, an orthogonal validation framework was employed. This was achieved utilizing gold-standard analytical reference materials, specifically the NA12878 genomic DNA standard and well-characterized External Quality Assessment (EQA) sample. For these samples, the exact genomic profile has been rigorously established and independently validated by the scientific community. The variant calling pipeline was executed on these reference samples both with and without the application of the upstream masking. The resulting variant call formats (VCFs) were then evaluated against the established Truth Sets. Variants that were uniquely called in the unmasked pipeline (residing within the LCRs) but were completely absent from the established high-confidence Truth Sets were classified as false-positive technical artifacts. This comparative benchmarking provided an independent, data-driven validation of the masking strategy’s specificity.

### 2.5. Comparative Variant Calling Analysis

To accurately assess the impact of the spatial masking strategy, a comparative variant calling analysis was performed. We analyzed our in-house sequenced analytical references (NA12878 and EQA)—both of which were independently sequenced utilizing both the hybridization-based and amplicon-based workflows—using our respective preprocessing pipelines, thus producing Binary Alignment Map (BAM) files from the raw FASTQ files. While the in-house analytical references and the AURORA cohort were processed from raw FASTQ files through the complete preprocessing and alignment workflow, the TCGA-BRCA cohort was obtained as pre-aligned BAM files from dbGaP and entered the analysis at the variant calling stage. Variant calling was subsequently executed in parallel under two distinct spatial constraints.

#### Variant Calling Strategies

An initial baseline analysis was executed utilizing the standard, manufacturer-provided ROIs defined by the original enrichment kit BED file. We used a parallel optimized analysis employing a targeted masking strategy restricted to the filtered ROI, ensuring the variant caller operated strictly within high-callability regions to effectively neutralize thermodynamic alignment ambiguity.

### 2.6. Hybridization-Based Pipeline

#### 2.6.1. Sequencing Data Preprocessing and Quality Control

Raw paired-end FASTQ files derived from the in-house whole-exome sequencing of both analytical references (Table 1) underwent initial quality control using FastQC (v0.12.1) [20]. Data preprocessing was performed with fastp (v0.24.0), including automated adapter clipping, a 15-bp 5’ hard clip, and a 3’ sliding-window quality filter (4-bp window, mean Phred < 20) [21]. Reads shorter than 50 bp were excluded. Because the TCGA-BRCA clinical cohort was acquired directly from dbGaP as pre-aligned BAM files, this initial FASTQ processing step was performed for the in-house datasets (Table 1) and AURORA cohort (Table 3). For the TCGA-BRCA cohort, the baseline exome target regions were restricted to the Broad Institute’s intervals list (exome_calling_regions.v1.interval_list; GRCh38), available via the Google Cloud Public Datasets repository (https://storage.googleapis.com/gcp-public-data--broad-references/hg38/v0/exome_calling_regions.v1.interval_list, accessed on 15 January 2026). This interval set represents the standardized sequencing targets used for the uniform processing of TCGA whole-exome sequencing (WES) data. For the AURORA cohort, the analysis was restricted to the exome target regions defined by the IDT xGen Exome Research Panel v1.0 BED file, corresponding to the WES capture design used for this dataset. The specific normal blood germline samples utilized in this study, along with their unique identifiers and corresponding alignment files, are detailed in Table 2 and Table 3.

#### 2.6.2. Alignment and Data Processing

Preprocessed reads were converted to uBAM format via GATK FastqToSam (v4.6.0.0) for Read Group assignment and aligned to the respective human reference genome (GRCh38.d1.vd1 or GRCh37/hs37d5), according to Table 1 and Table 3, using the Spark-enabled Burrows–Wheeler Aligner (BWA-MEM) within GATK (v4.6.0.0) [16,22,23]. Conversely, pre-aligned BAM files for the TCGA-BRCA cohort (aligned to GRCh38.d1.vd1.fa) were directly utilized (Table 2). Post-alignment processing followed GATK Best Practices [16,22], involving duplicate marking (MarkDuplicates), positional sorting (SortSamSpark), and Base Quality Score Recalibration (BQSRPipelineSpark) restricted to targeted capture intervals using cohort-specific BED files (Table 1 and Table 3) and known variant sites (dbSNP v138, Mills, 1000 Genomes gold standard INDELs and the known INDELs reference set) [22].

#### 2.6.3. Germline Variant Calling

Germline variant discovery was performed on the recalibrated BAM files using GATK HaplotypeCaller (v4.6.0.0) [22,24]. To optimize computational efficiency and minimize off-target false positives, variant calling was spatially restricted to the targeted genomic intervals defined by each cohort’s specific enrichment kit ROI (Table 1 and Table 2). To facilitate robust downstream filtering, extensive statistical annotations were embedded into the raw VCF files during the calling process. Specifically, the StandardAnnotation and StandardHCAnnotation modules were utilized, enriching the output with metrics (MQ, MQRankSum, ReadPosRankSum, FS, SOR, and DP). A minimum base quality score threshold of 20 and a Phred-scaled confidence threshold (QUAL) of 30.0 were enforced to ensure the initial detection of high-confidence alleles.

#### 2.6.4. Variant Quality Score Recalibration (VQSR) and Final Filtering

Variant Quality Score Recalibration (VQSR) was performed independently for SNPs and INDELs using the GATK VariantRecalibrator and ApplyVQSR tools. Standard truth and training sets (HapMap, Omni, 1000 Genomes, and Mills) were employed with a truth sensitivity threshold of 99.9% [22,24]. Only variants achieving a “PASS” filter status were retained for downstream analysis. The complete workflow, encompassing preprocessing, alignment, variant calling, and filtration steps, was executed via a custom bash script.

### 2.7. Amplicon-Based Pipeline

#### 2.7.1. Preprocessing and Alignment via GeneGlobe

Raw FASTQ files derived from the in-house amplicon-based sequencing of both analytical standards (NA12878 and EQA) were processed for targeted NGS data analysis and alignment utilizing the QIAGEN GeneGlobe Data Analysis Center (QIAGEN, Hilden, Germany) for the QIAseq Targeted DNA Pro Panel (PHS-001Z) (Table 1).

#### 2.7.2. Germline Variant Discovery and Refinement

Alignment-ready BAM files exported from GeneGlobe Data Analysis Center were utilized for germline variant discovery using GATK HaplotypeCaller (v4.6.0.0) [22,24]. Variant calling was strictly confined to the targeted panel ROI (Table 1). To minimize PCR-induced amplification artifacts inherent to amplicon sequencing, the –pcr-indel-model AGGRESSIVE parameter was employed. To prioritize high-confidence calls, a minimum base quality threshold of 20 and a Phred-scaled confidence threshold (QUAL) of 30.0 were enforced during variant discovery. Standard annotation metrics (StandardAnnotation, StandardHCAnnotation) were concurrently embedded to facilitate downstream quality assessment [22,24].

#### 2.7.3. Hard Filtering

Subsequent hard filtering was applied utilizing the GATK VariantFiltration module to exclude variants demonstrating a Quality by Depth (QD) < 2.0 or a QUAL < 30.0 [22,24]. Finally, only high-confidence variants passing all applied filters were retained for downstream analysis using the GATK SelectVariants tool [22,24]. The downstream variant discovery and hard filtering steps, following the export of alignment-ready BAM files from GeneGlobe, were implemented using a custom bash script.

### 2.8. Performance Evaluation and Functional Annotation

Performance quantification was executed based on cohort type. For the analytical references (NA12878 and EQA), exact coordinate matching was utilized to evaluate diagnostic sensitivity and precision. To accurately compute True Positives (TPs), False Positives (FPs), and False Negatives (FNs) while ensuring the discrete resolution of multiallelic sites, a composite variant identifier comprising the chromosome, genomic coordinate, and alternative allele (CHROM/POS/ALT) was extracted from the generated VCF files and utilized for all comparisons against the reference consensus. Conversely, performance evaluation for the clinical TCGA-BRCA and AURORA cohorts relied on the profiling of structural mutational signatures to distinguish biologically plausible variants from technical artifacts. Transition/transversion (Ti/Tv) ratios were calculated exclusively for biallelic SNPs, while Insertion/Deletion (Ins/Del) ratios were calculated for structural variants. Variants exhibiting extreme deviations from established whole-exome sequencing baselines were interpreted as technical noise, specifically: (1) Ti/Tv ratios falling significantly below the expected 2.8–3.0 threshold [16,25] and approaching the 0.5 ratio characteristic of systematic sequencing errors and alignment artifacts [16]; (2) Ins/Del ratios dropping substantially below 1.0. However, it is important to note that due to their inherent structural dynamics, specific LCRs may naturally exhibit biologically relevant deviations from these global genomic averages.

Functional annotation and clinical interpretation of the filtered artifacts were performed bioinformatically using the Ensembl Variant Effect Predictor (VEP v113.0) [26], deployed via a SingularityCE (v4.2.0) [27] container to ensure pipeline stability. The workflow integrated custom databases to cross-reference clinical significance (ClinVar release 20260329 [28]), and global annotation status via dbSNP inclusion rates.

### 2.9. Statistical Analysis and Data Visualization

All statistical metrics, including aggregate mean values, standard deviations and observed min–max ranges for cohort-level variant distributions, were calculated programmatically. For analytical reference standards, performance was quantified using true positives, false positives, false negatives, sensitivity/recall, precision, and Δ precision gains separately for SNPs and INDELs. For cohort-level analyses, statistical comparisons were performed across two independent WES cohorts: TCGA-BRCA hybrid-capture WES (*n* = 10) and AURORA-BRCA PCR-based capture WES (*n* = 15). Analyses were performed separately for each cohort and then summarized across the full combined dataset when cohort-level pooling was appropriate. To assess structural, allelic-balance, and annotational differences between LCR-masked retained calls and Unmasked-artifact excluded calls, paired sample-level comparisons were performed using the Wilcoxon signed-rank test, with two-tailed paired *t*-tests also reported for numerical summary tables where applicable. Statistical significance was defined at p<0.05.

For variant allele-frequency (VAF) analyses, pooled variant-level distributions were compared using Kolmogorov–Smirnov and Mann–Whitney U tests, while sample-level median VAF comparisons were assessed using paired tests. Figure significance brackets were based on paired Wilcoxon signed-rank tests. All statistical analyses were performed using Python (version 3.9) with pandas (version 2.3.3) [29], NumPy (version 2.0.2) and SciPy (version 1.13.1) [30].

Bioinformatics data manipulation and cross-referencing were executed in the R statistical environment (version 4.3; R Foundation for Statistical Computing, Vienna, Austria) utilizing the dplyr, tidyr [31], and GenomicRanges packages [32]. Complex data visualizations, including KDE density plots, violin/boxplot distributions, cohort-stratified bar plots, and formatted log-scale annotation charts, were generated using Python (version 3.9; Python Software Foundation, Wilmington, DE, USA) with pandas (version 2.3.3) [29], matplotlib (version 3.9.4) [33] and seaborn (version 0.13.2), alongside R’s ggplot2 framework (version 4.0.3) [31].

## 3. Results

To comprehensively evaluate the efficacy and clinical safety of the targeted spatial masking strategy, our analysis was conducted in two primary phases. First, we established the analytical validity of the protocol by measuring diagnostic sensitivity and precision gains across highly validated reference standards (NA12878 and EQA). Second, we applied the spatial filtration framework to two clinical whole-exome sequencing (WES) cohorts (TCGA-BRCA, AURORA) to assess the restoration of physiological mutational signatures and profile the clinical annotation status of the filtered artifacts. The following subsections detail the quantitative and structural impact of removing this locus-specific technical noise.

### 3.1. Preservation of Diagnostic Sensitivity and Artifact Mitigation

**Figure 1:**
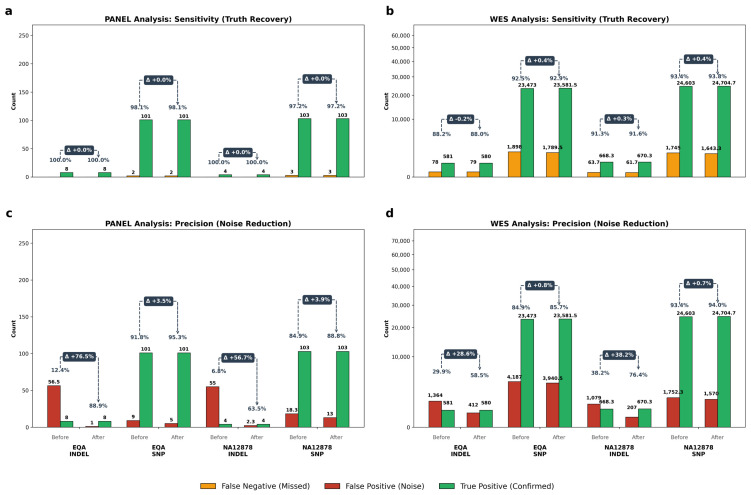
Impact of Spatial Masking on Amplicon-Based capture Targeted Panel and Hybrid-Capture WES. Comparative performance metrics for analytical reference standards (EQA and NA12878). (**a**,**b**) Spatial masking achieved a 100% True Positive (green) retention rate across both amplicon-based and hybrid-capture architectures, ensuring zero loss of diagnostic sensitivity. Notably, a marginal increase in True Positives (green) was observed in the WES cohort, representing approximately 100 rescued SNPs. (**c**,**d**) Important reduction in False Positive calls (red) resulted in significant precision gains, most prominently for INDELs within the EQA and NA12878 targeted panels, with corresponding improvements in WES datasets. All reported counts and percentages represent the mean of technical replicates (NA12878, *n* = 3; EQA, *n* = 2). Sensitivity and Precision gains are expressed as delta (Δ) values, representing the percentage-point improvement compared to the unmasked callset (Before; After). True Positive: variants called by the variant caller as the same genotype as the reference data (EQA/NA12878). False Positive: variants called by the variant caller but not in the reference set (EQA/NA12878). False Negative: reference standard (EQA/NA12878) variants that were not called. Precision: TP/(TP + FP); Sensitivity: TP/(TP + FN).

To establish the analytical validity of the targeted spatial masking strategy, we first benchmarked the protocol against highly validated analytical reference standards (NA12878 and EQA reference standards) across both amplicon-based targeted panels and hybrid-capture WES architectures (Figure 1). The primary objective was to filter technical noise without compromising the detection of genuine biological variants. The reported results represent the mean values across technical replicates (triplicate for NA12878; duplicate for EQA). The masking protocol demonstrated stable variant calling accuracy by maintaining a 100% True Positive retention rate and introducing zero False Negatives across the targeted panel assays. Furthermore, the targeted exclusion of LCRs resulted in the reduction in False Positives, yielding significant gains in analytical precision. This improvement was most pronounced in INDEL calling, driving a +76.5% precision increase for EQA and +56.7% for NA12878 in the panel data (denoted as Δ values in Figure 1). Similar precision gains (+28.6% and +38.2%, respectively) were observed in the WES data, alongside the safe retention of approximately 100 True Positive SNPs that might otherwise have been lost due to artifactual noise. This analysis confirmed that the LCR-associated calls generated by the unmasked pipeline were overwhelmingly absent from the biological ground truth, classifying them directly as deterministic mapping errors. These robust baseline metrics confirm that targeted spatial masking safely filters systematic false-positive sequencing artifacts, justifying the protocol’s subsequent application to the broader clinical WES cohorts.

### 3.2. Restoration of Biological Signatures and Structural Integrity

**Figure 2:**
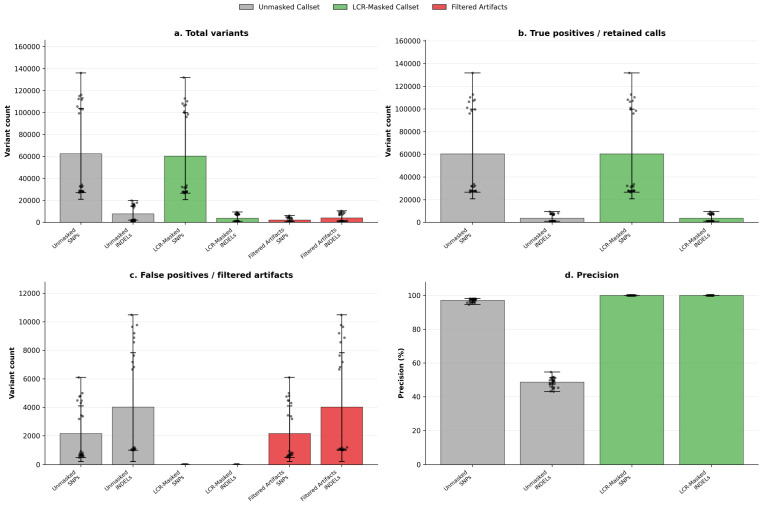
Integrated variant recovery and precision profile after selective LCR masking across TCGA-BRCA and AURORA cohorts. Multi-panel visualization of cohort-level variant recovery and precision metrics across TCGA-BRCA hybrid-capture WES samples and AURORA PCR-based capture WES samples (combined *n* = 25). (**a**) Total variant counts in the Unmasked callset, LCR-Masked callset, and Filtered artifact fraction. (**b**) True-positive retained calls in the Unmasked and LCR-masked callsets. (**c**) False-positive artifact-excluded calls in the Unmasked callset, LCR-masked callset, and Filtered artifact fraction. (**d**) Precision before and after LCR masking for SNPs and INDELs. Bars indicate mean values, error bars indicate ± SD, thin black whiskers indicate observed min–max ranges, and dots represent individual samples. The wider dispersion in absolute variant counts reflects the integration of two independent cohorts with different baseline callset scales and enrichment designs.

To evaluate the real-world clinical utility and cross-cohort reproducibility of the masking protocol, we analyzed 25 normal blood WES samples from two independent breast cancer cohorts: 10 hybrid-capture WES samples from TCGA-BRCA and 15 PCR-based capture WES samples from AURORA cohort. The baseline Unmasked callsets revealed a substantial burden of artifact excluded calls associated with low-complexity regions, particularly among INDELs. Prior to masking, Unmasked callsets showed reduced variant-level precision, with this effect being most pronounced for INDELs. Implementation of the targeted upstream LCR-masking strategy reduced the artifact fraction while preserving the retained biological callset, resulting in complete operational precision within the LCR-Masked callset framework for both SNPs and INDELs (Table 4, Figure 2).

Across the combined cohort, baseline SNP precision was 97.07% ± 1.02%, whereas baseline INDEL precision was substantially lower at 48.62% ± 2.78%. After LCR masking, the Filtered fraction accounted for 2157 ± 1939 SNP artifacts and 4026 ± 3808 INDEL artifacts per sample. These findings indicate that upstream spatial masking preferentially removes the low-complexity artifact burden while preserving the core biological signal. The expanded numerical summary, including mutational signatures, dbSNP annotation, statistical testing, and cohort-stratified results, is provided in Appendix A, as well as Table A1 and Table A2.

### 3.3. Suppression of Pseudo-Multiallelic Artifacts and Clinical Validation of Diagnostic Safety

**Figure 3:**
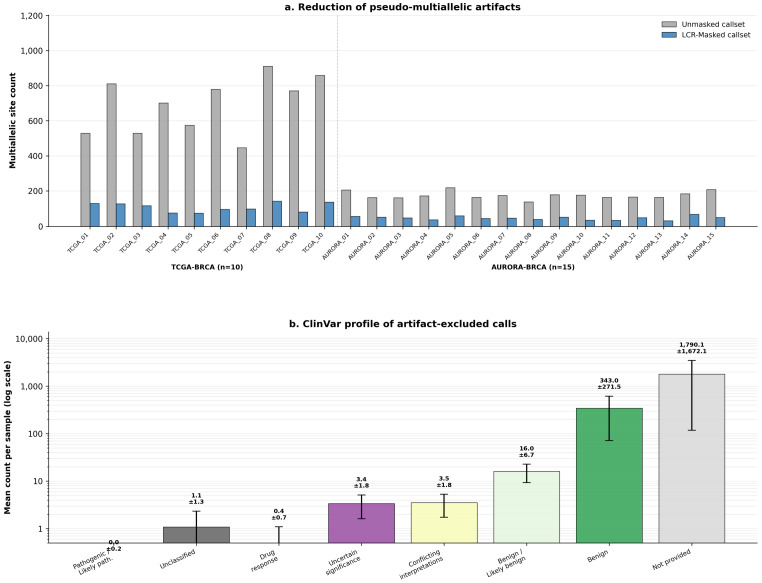
Suppression of Pseudo-Multiallelic Artifacts and Clinical Profiling across TCGA-BRCA and AURORA cohorts. (**a**) Bar chart representing the absolute counts of multiallelic sites before and after targeted upstream LCR masking across TCGA-BRCA and AURORA samples (combined *n* = 25). The Unmasked callset (grey) shows recurrent multiallelic positions consistent with alignment ambiguity in low-complexity regions, whereas LCR-masked callsets (blue) show marked reduction in these artifacts across both cohorts. (**b**) ClinVar annotation profile of excluded artifact calls. Values represent mean count per sample ± SD on a log-scaled y-axis. The excluded fraction was dominated by variants annotated as not provided, benign, benign/likely benign, uncertain significance, or conflicting interpretation, while pathogenic/likely pathogenic disease-associated annotations were negligible at the cohort level. Sample aliases used for compact visualization in panel a correspond to the TCGA-BRCA and AURORA WES data; identifiers listed in Table 2 and Table 3.

Comprehensive structural profiling demonstrated that low-complexity-associated noise was highly prone to generating alignment ambiguity and pseudo-multiallelic artifacts. In the baseline Unmasked callsets, both TCGA-BRCA and AURORA samples showed recurrent multiallelic positions consistent with overlapping artifactual calls within repetitive or poorly mappable sequence contexts. Application of targeted upstream LCR masking markedly reduced these pseudo-multiallelic sites across the combined cohort, indicating that spatial exclusion of low-complexity intervals streamlines downstream variant representation and annotation (Figure 3a).

To evaluate the diagnostic safety of the excluded call fraction, excluded artifacts were cross-referenced against ClinVar annotations. The ClinVar profile of the excluded fraction was dominated by variants annotated as not provided, benign, benign/likely benign, uncertain significance, or conflicting interpretation, whereas pathogenic/likely pathogenic disease-associated annotations were negligible at the cohort level (Figure 3b). This supports the interpretation that the masking protocol preferentially removes technical noise rather than clinically actionable disease-associated pathogenic variation. A small subset of excluded artifacts carried drug-response annotations, highlighting an important distinction between diagnostic pathogenicity and pharmacogenomic annotation. Although these variants are not inherently diagnostic of disease pathogenesis, their localization within low-complexity regions emphasizes a known limitation of short-read sequencing for pharmacogenomic loci, where targeted orthogonal validation may be required for therapeutic decision-making.

### 3.4. VAF Signatures Differentiate Mendelian Inheritance Patterns from Alignment Artifacts

**Figure 4:**
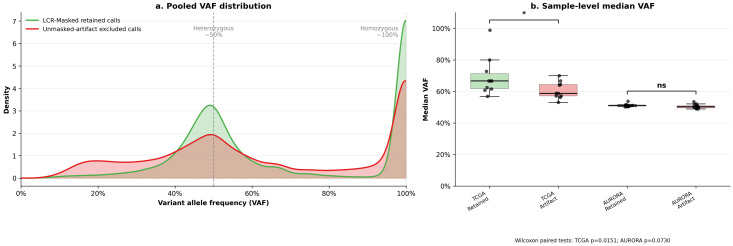
Variant allele-frequency profiling of retained and artifact-enriched calls across TCGA-BRCA and AURORA cohorts, (**a**) Pooled variant allele frequency (VAF) density plot visualized using kernel density estimation (KDE), comparing LCR-masked retained calls with Unmasked-artifact excluded calls across the combined cohort. Vertical dashed lines indicate the expected germline heterozygous and homozygous VAF regions at approximately 50% and 100%, respectively. Retained calls showed stronger enrichment around canonical germline VAF peaks, whereas artifact excluded calls showed broader dispersion across the allele-frequency spectrum, including increased representation at lower and intermediate VAF values. (**b**) Sample-level median VAF comparison stratified by cohort and call category. Boxes represent per-sample median VAF distributions, and individual points represent samples. Significance brackets indicate paired within-cohort comparisons between retained and artifact calls using Wilcoxon signed-rank tests. TCGA-BRCA showed a significant VAF shift (* *p* = 0.0151), whereas AURORA showed a non-significant trend in the same direction (ns, *p* = 0.0730). Across the combined cohort, the paired median VAF difference was significant (*p* = 0.0027).

Analysis of the Variant Allele Frequency (VAF) was used to further evaluate whether the retained and excluded call fractions showed distinct allelic-balance behavior across the combined TCGA-BRCA and AURORA cohort. In the pooled density analysis, LCR-masked retained calls showed strong enrichment around the expected Mendelian inheritance patterns, exhibiting prominent density peaks at ∼0.5 (heterozygous) and ∼1.0 (homozygous) allelic fractions (Figure 4a). In contrast, Unmasked-artifact excluded calls displayed a broader VAF distribution, with increased representation across lower and intermediate allele-frequency ranges and a less sharply defined canonical germline profile. This pattern supports the interpretation that upstream LCR masking preferentially removes unstable calls associated with low-complexity alignment ambiguity rather than uniformly depleting high-confidence germline variation. Sample-level median VAF analysis confirmed a significant difference between retained and artifact-excluded calls in the combined cohort. The effect was significant in TCGA-BRCA, where retained calls showed higher median VAF than Unmasked-artifact excluded calls (Wilcoxon signed-rank test, *p* = 0.0151), whereas AURORA showed a non-significant trend in the same direction (*p* = 0.0730) (Figure 4b). Across the combined cohort, the paired sample-level median VAF difference was significant (Wilcoxon *p* = 0.0027). These results indicate that the artifact-enriched fraction has a distinct allelic-balance profile, while also demonstrating that the magnitude of the VAF shift varies by cohort and capture design.

### 3.5. The dbSNP Paradox and Deterministic Noise Profiling

**Figure 5:**
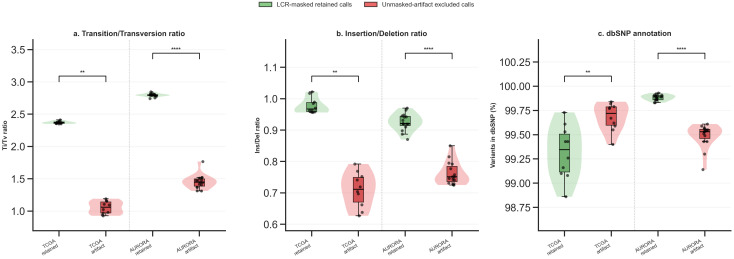
Cohort-stratified mutation-signature distortion and dbSNP annotation after selective LCR masking. Multi-panel visualization comparing LCR-masked retained calls (green) with Unmasked-artifact excluded calls (red) across TCGA-BRCA and AURORA cohorts. (**a**) Transition/transversion (Ti/Tv) ratios show that retained calls preserve expected biological substitution balance, whereas artifact-excluded calls show strong distortion in both cohorts. (**b**) Insertion/deletion (Ins/Del) ratios similarly demonstrate structural distortion in the artifact-excluded fraction relative to retained calls. (**c**) dbSNP annotation rates show cohort-dependent behavior: TCGA-BRCA artifact-excluded calls show higher dbSNP annotation rate than retained calls, whereas AURORA shows the opposite direction. Boxplots indicate median and interquartile range, violin densities illustrate the sample-level distribution, and individual points represent samples. Brackets indicate paired sample-level Wilcoxon signed-rank tests within each cohort. ** *p* < 0.01; **** *p* < 0.0001. Complete numerical summaries, including mean ± SD and observed ranges, are provided in Appendix A, as well as Table A1 and Table A2.

To systematically demonstrate the deterministic nature of the excluded artifacts, we contrasted the multidimensional quality signatures of LCR-masked retained calls with Unmasked-artifact excluded calls across TCGA-BRCA and AURORA cohorts. As expected in both cohorts, retained calls maintained stable biological mutational profiles, whereas the artifact-excluded fraction showed marked distortion of transition/transversion (Ti/Tv) and insertion/deletion (Ins/Del) balance (Figure 5a,b). In TCGA-BRCA, the artifact-excluded fraction showed a depressed Ti/Tv ratio for SNPs (1.054 ± 0.098) and a reduced Ins/Del ratio for INDELs (0.710 ± 0.055). The AURORA cohort showed the same directional distortion, with retained calls preserving higher Ti/Tv ratios (2.80 ± 0.03) and more physiological Ins/Del balance (0.93 ± 0.03) than the artifact-excluded fraction (Ti/Tv: 1.46 ± 0.11; Ins/Del: 0.76 ± 0.04). Paired sample-level Wilcoxon testing confirmed significant differences in both cohorts, supporting the interpretation that low-complexity-associated artifacts represent a reproducible and non-random class of technical noise.

Analysis of dbSNP inclusion rates revealed a cohort-dependent pattern rather than a uniform pooled effect in variant annotation. In TCGA-BRCA cohort, retained calls exhibited an expected dbSNP annotation rate of 99.32% (± 0.27%). However, the artifact-excluded calls showed higher database inclusion rate (99.68% ± 0.14%; *p* = 0.002; Figure 5c) than retained calls, reproducing the original dbSNP paradox and suggesting that some recurrent low-complexity artifacts may have been historically catalogued in public variant databases. In contrast, AURORA cohort showed the opposite directional pattern, with retained calls reaching the 99.88% (± 0.03%) dbSNP annotation rate compared to the lower rate of the artifact-excluded fraction (99.49% ± 0.12%; *p* < 0.0001; Figure 5c). Consequently, the combined dbSNP comparison was not significant, despite significant within-cohort differences in opposite directions. These findings indicate that dbSNP presence alone should not be interpreted as a universal marker of variant authenticity. Instead, database annotation should be evaluated alongside sequence context, cohort design, and orthogonal quality metrics such as Ti/Tv ratio, Ins/Del balance, VAF behavior, and spatial localization within low-complexity regions. Larger-scale cohort validations are warranted to determine the full extent and generalizability of this cohort-dependent annotation effect.

## 4. Discussion

The presence of systematic sequencing errors within LCRs is translated directly into a bioinformatic bottleneck, as variant calling algorithms struggle to differentiate true biological variations from mapping-induced artifacts, while the maintenance of an appropriate equilibrium between artifact management and the retention of pathogenic variants remains a critical challenge in clinical genomics. Our results demonstrate that targeted spatial masking of low-complexity regions (LCRs) prior to variant calling effectively alleviates this computational bottleneck for variants residing outside of these repetitive elements. However, we do not yet know the exact extent to which these LCRs may have clinical relevance. Much like smRNAs and microproteins, which have been largely neglected but have recently gained traction, LCRs may still turn out to be highly relevant, and our masking strategy would inherently hamper the detection of future LCR-associated variants. While this limitation makes little difference when using standard short-read technologies (e.g., Illumina) where resolving ambiguities remains difficult, emerging longer reads (e.g., PacBio) may avoid this problem and provide potentially useful information that could require a paradigm shift. Thus, our approach is primarily a highly useful tool for the accurate detection of variants residing outside LCRs, which represents the standard clinical use today.

By applying this strategy across clinical reference panels and two independent breast cancer WES cohorts, including TCGA-BRCA hybrid-capture WES and AURORA PCR-based capture WES, we filtered thousands of deterministic artifact-excluded calls while supporting the retention of the core biological callset. ClinVar profiling of the excluded artifact fraction showed that pathogenic/likely pathogenic disease-associated annotations were negligible [34] at the cohort level, supporting the diagnostic safety of the spatial filtration parameters across the analyzed datasets. Mitigating the loss of clinical findings is critical for clinical implementation, as it addresses the persistent concern regarding potential false negatives in challenging regions, areas often considered bioinformatic ’blind spots’ where clinical variants and technical artifacts frequently overlap [35].

While the exclusion of problematic genomic regions is a recognized concept in genomics, the practical advantage of our approach lies fundamentally in the timing of the bioinformatic intervention and its precision. As outlined above, established resources, such as the GIAB difficult-region stratifications [15,17] or comprehensive ENCODE blacklists [14], are predominantly applied post-variant calling for validation, algorithmic benchmarking, and VCF intersection. In these traditional post-calling workflows, variant algorithms must still invest substantial computational resources attempting to resolve severe thermodynamic ambiguities within LCRs, generating complex VCF clutter that must be subsequently filtered by statistical methods like VQSR [16]. Instead of relying solely on downstream filtering to manage this noise, our targeted spatial masking operates upstream to bulletproof the pipeline. By preemptively blinding the variant caller to simple hyper-mutable regions, this upstream intervention acts synergistically with established downstream filters: it simplifies the bioinformatic workflow, protects statistical models from being saturated by artifactual noise, and significantly reduces VCF clutter, providing a streamlined solution highly suitable for single-sample clinical diagnostics.

The comprehensive signature profiling supports the artifact-enriched status of the excluded fraction and indicates that these calls represent structured, low-complexity-associated technical noise rather than random loss of biological variation. Across two independent breast cancer WES cohorts with distinct capture designs, the Unmasked-artifact excluded fraction showed the same directional distortion of Ti/Tv and Ins/Del profiles, whereas LCR-masked retained calls preserved more physiologic mutational balance. This reproducibility suggests that low-complexity-associated artifacts represent a structured and non-random class of technical noise generated by sequence-context-dependent alignment ambiguity. The distorted artifact profiles also fall below commonly expected whole-exome sequencing baselines, where Ti/Tv ratios typically range from 2.8 to 3.0 [16,25] and Ins/Del ratios approximate 1.0. However, it is important to note that these global baseline metrics may not uniformly apply to all genomic landscapes. Specific LCRs, such as Alu elements or regions harboring ancient retroviral DNA, possess distinct nucleotide compositions and may naturally display dissimilar mutational signatures [36,37].

Furthermore, the suppression of pseudo-multiallelic clutter directly corroborates the benchmarking analyses by Heng Li [5] and Schirmer et al. [38], who demonstrated that polymerase slippage within homopolymers inherently generates severe thermodynamic alignment ambiguities. It should also be noted that while our overall profiling demonstrates robust alignment with expected Mendelian patterns, deviations may naturally occur, especially in somatic mutations with varying degrees of healthy and affected tissue composition, or in cases of chimeric individuals and mosaicism [39]. Additionally, while polymerase slippage frequently generates technical noise, it can also occur naturally with significant clinical relevance, such as the FMR1 repeat expansions in Fragile X syndrome. Indeed, functional patterns of LCRs have been identified across numerous human genes [40,41]. Therefore, in clinical scenarios where LCR-associated diseases or complex mosaicism are suspected, targeted masking must be complemented with dedicated variant detection assays. Targeted spatial exclusion maintains the accuracy of variant calls in flanking regions, effectively addressing the signal interference inherent to Low-Complexity Regions (LCRs).

A key finding of the expanded analysis is that the originally observed “dbSNP paradox” should now be interpreted as a cohort-dependent annotation phenomenon rather than a uniform pooled effect. The TCGA-BRCA pattern remains compatible with the original dbSNP paradox, whereas the AURORA cohort demonstrates that this behavior is not universal and may depend on cohort structure, capture design, local sequence composition, or database representation of unstable loci. This context-dependent saturation effect creates a potential vulnerability in clinical pipelines, suggesting a layer of complexity in modern variant interpretation. This observation remains compatible with recent reports identifying “camouflaged” genomic regions that consistently generate reproducible, deterministic mapping errors across diverse sequencing technologies [8]. Because these LCR-induced artifacts are driven by underlying genomic architecture and generic probe design, they may be repeatedly mischaracterized as true biological variation and cataloged into public repositories [9].

Importantly, it is equally plausible that the natural thermodynamic instability of these regions drives genuine, hyper-variable polymorphism across human populations, which is accurately reflected in public databases. Regardless of whether this saturation is driven by widespread technical mischaracterization, genuine biological hyper-mutability, or cohort-specific annotation structure, this phenomenon indicates that isolated database annotation is insufficient as a primary metric for variant authentication.

## 5. Conclusions

In conclusion, resolving the persistent bioinformatics bottleneck caused by LCRs through spatial masking is highly effective for detecting non-LCR-associated germline variants, though it inherently comes at the cost of missing potentially yet uncharacterized variants residing within the masked regions. In addition to continuous algorithmic improvements in variant calling, recent global benchmarking efforts, such as the precisionFDA Truth Challenge V2, emphasize that LCRs remain the primary frontier for sequence accuracy [19]. The cohort-dependent dbSNP behavior observed here reveals a challenge in variant annotation: database presence alone cannot reliably distinguish biological variation from recurrent low-complexity-associated artifacts. A practical resolution involves implementing upstream noise suppression within the bioinformatics workflow while interpreting database annotation together with sequence context, cohort design, VAF behavior, and mutational-signature metrics. Our findings demonstrate that targeted spatial masking of low-complexity regions provides a robust clinical-level strategy for reducing this persistent bioinformatic bottleneck. Locus-specific exclusion of systematic artifacts supports the retention of diagnostic sensitivity and inherent mutational signatures while simplifying the manual curation workflow. Ultimately, implementing this masking strategy as a foundational step in standard clinical workflows can improve the accuracy of variant interpretation and facilitate more reliable patient diagnostics.

## Figures and Tables

**Table 1:** Analytical reference cohort specifications, enrichment strategies, and corresponding computational reference assemblies.

Sample/Cohort	Sequencing Assay	Enrichment Kit (Target ROI)	Reference Assembly
EMQN Targeted Panel	Amplicon Targeted Panel	QIAseq PHS-001Z	GRCh38.d1.vd1.fa
EMQN WES	Hybridization WES	Twist ILMN Exome 2.5 Plus	hs37d5.fa (GRCh37)
NA12878 Targeted Panel	Amplicon Targeted Panel	QIAseq PHS-001Z	GRCh38.d1.vd1.fa
NA12878 WES	Hybridization WES	Twist ILMN Exome 2.5 Plus	hs37d5.fa (GRCh37)

All analytical standard libraries were sequenced utilizing the Illumina NextSeq 2000 platform. To rigorously assess technical reproducibility, the NA12878 reference standard was processed and sequenced in triplicate, while the EMQN clinical reference standard was processed in duplicate.

**Table 2:** TCGA-BRCA cohort sample identifiers, database UUIDs, and corresponding alignment file names utilized for the clinical optimization cohort.

Sample Alias	Entity UUID	BAM File Name
TCGA_S1	58a05450-0f93-4d4e-a53b-0d8e467b9946	46160082-58d4-47f5-a49a-505ca987fd79_wxs_gdc_realn.bam
TCGA_S2	d3f6046d-db47-41c7-93c2-91c4991b69c4	57b5183f-1ef3-4f14-981e-2ecf416c387f_wxs_gdc_realn.bam
TCGA_S3	244d53de-31c9-4700-a9e3-289482b9bd58	60b54869-5add-4799-89a0-e5f090801826_wxs_gdc_realn.bam
TCGA_S4	632182ba-0940-4e6d-91df-425dcffbb880	7fd1a063-e742-4ebc-acbd-c81ba11f1cc3_wxs_gdc_realn.bam
TCGA_S5	cbbc10e5-8356-4dab-974b-78a9c3910ec5	9b5d1a2f-ccca-45de-b995-85754015b39c_wxs_gdc_realn.bam
TCGA_S6	db6544dc-566a-4502-9601-e6d2b002cd4d	a9ac6ea6-e114-4fac-8372-86cb23c835ec_wxs_gdc_realn.bam
TCGA_S7	e8990686-bdce-403e-be1e-aada2cc739dc	b0e8676c-6f2d-4691-96c5-b323dded9687_wxs_gdc_realn.bam
TCGA_S8	4aae9444-58fd-430f-afcc-d08755eda3e2	bd560d56-fd13-4213-8e29-2a99fee0a055_wxs_gdc_realn.bam
TCGA_S9	0ae99282-6f6c-4c11-b242-bc363a2aada2	dd6007264-3412-46b3-8775-f2c42146de20_wxs_gdc_realn.bam
TCGA_S10	b41ee0d2-5122-4cdc-9e70-cba60b9e96ed	f1681b14-25e1-4bc6-a97d-18928662d45b_wxs_gdc_realn.bam

All samples were processed utilizing Whole-Exome Sequencing (WES). Alignments were performed against the GRCh38.d1.vd1.fa reference assembly, with target capture defined by the GDC Standard Exome Targets enrichment kit.

**Table 3:** Aurora US Metastatic Breast Cancer Retrospective Project cohort sample identifiers, sample names and corresponding SRR Run accession codes utilized for the clinical optimization cohort.

Sample Alias	Sample Name	Run
AUR_S1	AUR-AE6X-NB1-A-1-0-D-A738-43	SRR21068524
AUR_S2	AUR-AERW-NB1-A-1-0-D-A738-43	SRR21068697
AUR_S3	AUR-AERX-NB1-A-1-0-D-A738-43	SRR21068539
AUR_S4	AUR-AERY-NB1-A-1-0-D-A738-43	SRR21068711
AUR_S5	AUR-AF94-NB1-A-1-0-D-A738-43	SRR21068551
AUR_S6	AUR-AF95-NB1-A-1-0-D-A738-43	SRR21068483
AUR_S7	AUR-AF99-NB1-A-1-0-D-A738-43	SRR21068706
AUR_S8	AUR-AFKF-NB1-A-1-0-D-A739-43	SRR21068597
AUR_S9	AUR-AFSO-NB1-A-1-0-D-A739-43	SRR21068726
AUR_S10	AUR-AFUI-NB1-A-1-0-D-A739-43	SRR21068522
AUR_S11	AUR-AFUN-NB1-A-1-0-D-A739-43	SRR21068693
AUR_S12	AUR-AG0J-NB1-A-1-0-D-A739-43	SRR21068721
AUR_S13	AUR-AG0M-NB1-A-1-0-D-A739-43	SRR21068718
AUR_S14	AUR-AG0N-NB1-A-2-0-D-A739-43	SRR21068560
AUR_S15	AUR-AG12-NB1-A-1-0-D-A739-43	SRR21068702

All samples were processed using Whole-Exome Sequencing (WES). Alignments were performed against the GRCh38.d1.vd1.fa reference assembly, with target regions defined by the IDT xGen Exome Research Panel v1.0 BED file, corresponding to the exome capture design used for the AURORA cohort.

**Table 4:** Effect of selective LCR masking on cohort-level variant recovery and precision.

Filtration Stage	Variant Type	Total Variants	TPs	FPs	Precision
Unmasked Callset	SNPs	62,488±41,417	60,331±39,567	2157±1939	97.07%±1.02%
	INDELs	7702±7121	3676±3352	4026±3808	48.62%±2.78%
LCR-masked Callset	SNPs	60,331±39,567	60,331±39,567	0 (Ref.)	100.00%±0.00%
	INDELs	3676±3352	3676±3352	0 (Ref.)	100.00%±0.00%
Filtered Artifacts	SNPs	2157±1939	–	2157±1939	–
	INDELs	4026±3808	–	4026±3808	–

Data are presented as mean ± SD across TCGA-BRCA hybrid-capture WES samples and AURORA PCR-based capture WES samples (combined *n* = 25). True positives (TPs) retained biological calls in the comparative masking framework; false positives (FPs) artifact-excluded calls filtered out by the spatial masking protocol. The Unmasked callset represents variant discovery using the original cohort-specific regions of interest without spatial filtration. The LCR-Masked callset represents the optimized callset generated by restricting variant discovery to high-complexity intervals prior to variant calling and recalibration. Filtered artifacts represent calls enriched within simple repeats and homopolymers removed by the targeted spatial masking strategy. Expanded numerical summaries, including mutational signatures, dbSNP annotation, statistical testing, and cohort-stratified analyses, are provided in Appendix A, Table A1 and Table A2.

## Data Availability

The genomic datasets analyzed during the current study are available through public repositories and commercial vendors as follows: The TCGA-BRCA normal blood whole-exome sequencing dataset is available in the database of Genotypes and Phenotypes (dbGaP) under accession number phs000178.v11.p8. The results published here are in whole or part based upon data generated by The Cancer Genome Atlas managed by the NCI and NHGRI. Information about TCGA can be found at http://cancergenome.nih.gov, (accessed on 14 November 2025). The AURORA US Metastatic Breast Cancer Retrospective Project normal blood whole-exome sequencing dataset is available in dbGaP under accession number phs002622.v1.p1. The analytical reference standard DNA NA12878 (Lot N84, CEPH/UTAH PEDIGREE 1463) is commercially available from the Coriell Institute for Medical Research (https://www.coriell.org/0/Sections/Search/Sample_Detail.aspx?Ref=NA12878&Product=DNA, accessed on 10 September 2025). Assembly-specific stratification files for homopolymers are available in the Genome in a Bottle (GIAB) v3.0 repository. The External Quality Assessment (EQA) diagnostic DNA dataset (scheme ’DNA SEQUENCING-NGS (Germline SNVs and INDELs)’) was obtained commercially from the European Molecular Genetics Quality Network (EMQN). Due to licensing and user agreements, the raw EQA data cannot be shared publicly but can be accessed by registered participants via the EMQN portal (https://www.emqn.org accessed on January 2024). The custom bioinformatics pipelines, including the bash scripts for filtering of the ROI, preprocessing, sequence alignment, variant calling and clinical interpretation are publicly available in the GitHub repository (https://github.com/Tremoulis/bigen_LCR_masking, created 19 June 2026).

## Appendix A

### Appendix A.1. Expanded Cohort-Level Variant Quality Metrics

Table A1 reports the TCGA-BRCA hybrid-capture WES cohort (*n* = 10), whereas Table A2 reports the AURORA WES cohort (*n* = 15). For each cohort, the tables summarize absolute variant counts, retained biological calls, artifact-enriched calls, precision, mutational signatures, dbSNP annotation rates, statistical significance, and observed ranges before and after targeted upstream LCR masking. Data are presented as mean ± SD [min–max]. The Unmasked callset refers to variant discovery using the original cohort-specific regions of interest without spatial filtration. The LCR-masked callset refers to the optimized callset generated by restricting variant discovery to high-complexity intervals prior to variant calling and recalibration. Filtered artifacts represent excluded sequencing and alignment noise enriched within repetitive elements and homopolymers. Statistical comparisons were performed using two-tailed paired *t*-tests comparing retained biological calls with the filtered artifact fraction within each cohort. Because dbSNP annotation showed cohort-dependent behavior, dbSNP values are interpreted as cohort-stratified quality descriptors.

**Table A1:** Expanded TCGA-BRCA cohort-level impact of selective LCR masking on variant quality, mutational signatures, and dbSNP annotation.

Metric	Unmasked Callset	LCR-Masked Callset	Filtered Artifacts
**SNPs**			
Total Variants	111,552±10,385 [99,232–136,117]	107,163±10,245 [96,045–131,826]	4389±884 [3187–6096]
True Positives	107,163±10,245 [96,045–131,826]	107,163±10,245 [96,045–131,826]	–
False Positives	4389±884 [3187–6096]	0±0 [0–0] (Reference)	4389±884 [3187–6096]
Precision	96.05%±0.79% [94.58–97.04%]	100.00%±0.00% [100.00–100.00%]	–
Mutational Signature	Ti/Tv =2.291±0.022 [2.262–2.334]	Ti/Tv =2.372±0.020 [2.350–2.412]	Ti/Tv =1.054±0.098 [0.927–1.191] ***
dbSNP Annotation	99.36%±0.26% [98.92–99.74%]	99.32%±0.27% [98.86–99.73%]	99.68%±0.14% [99.40–99.84%] ***
**INDELs**			
Total Variants	16,141±1815 [13,536–19,868]	7653±810 [6718–9379]	8488±1342 [6667–10,489]
True Positives	7653±810 [6718–9379]	7653±810 [6718–9379]	–
False Positives	8488±1342 [6667–10,489]	0±0 [0–0] (Reference)	8488±1342 [6667–10,489]
Precision	47.59%±3.98% [43.06–54.66%]	100.00%±0.00% [100.00–100.00%]	–
Mutational Signature	Ins/Del =0.829±0.037 [0.762–0.891]	Ins/Del =0.978±0.025 [0.956–1.022]	Ins/Del =0.710±0.055 [0.627–0.792] ***
dbSNP Annotation	99.36%±0.26% [98.92–99.74%]	99.32%±0.27% [98.86–99.73%]	99.68%±0.14% [99.40–99.84%] ***

Data are presented as mean ± SD [min–max]. TPs, true positives/retained biological calls in the comparative masking framework; FPs, false positives/artifact-enriched calls excluded by the spatial masking protocol; Ti/Tv, transition/transversion ratio; Ins/Del, insertion/deletion ratio; SD, standard deviation. Statistical significance was evaluated using two-tailed paired *t*-tests comparing retained biological calls with the filtered artifact fraction within each cohort. *** *p* < 0.0001.

**Table A2:** Expanded AURORA cohort-level impact of selective LCR masking on variant quality, mutational signatures, dbSNP annotation, and raw statistical values.

Metric	Unmasked Callset	LCR-Masked Callset	Filtered Artifacts
**SNPs**			
Total Variants	29,779 ± 2347 [27,014–34,231]	29,110 ± 2328 [26,519–33,591]	668 ± 110 [495–916]
True Positives	29,110 ± 2328 [26,519–33,591]	29,110 ± 2328 [26,519–33,591]	–
False Positives	668±110 [495–916]	0 (Ref.)	668±110 [495–916]
Precision	97.75%±0.39% [96.79–98.17%]	100.00%±0.00% [100.00–100.00%]	–
Mutational Signature	Ti/Tv =2.750±0.026 [2.690–2.800]	Ti/Tv =2.795±0.027 [2.741–2.847]	Ti/Tv =1.455±0.110 [1.312–1.767] ***
dbSNP Annotation	99.86%±0.03% [99.82–99.91%]	99.88%±0.03% [99.83–99.93%]	99.49%±0.12% [99.14–99.61%] ***
Raw *p*-values	–	–	Ti/Tv: *p* = 1.1515 $\times 10^{-16}$; dbSNP: *p* = 5.6539 $\times 10^{-9}$
**INDELs**			
Total Variants	2076±137 [1928–2356]	1024±87 [914–1164]	1051±58 [990–1192]
True Positives	1024±87 [914–1164]	1024±87 [914–1164]	–
False Positives	1051±58 [990–1192]	0 (Ref.)	1051±58 [990–1192]
Precision	49.31%±1.35% [47.41–51.74%]	100.00%±0.00% [100.00–100.00%]	–
Mutational Signature	Ins/Del =0.839±0.020 [0.802–0.879]	Ins/Del =0.926±0.028 [0.870–0.970]	Ins/Del =0.762±0.037 [0.725–0.850] ***
dbSNP Annotation	99.86%±0.03% [99.82–99.91%]	99.88%±0.03% [99.83–99.93%]	99.49%±0.12% [99.14–99.61%] ***
Raw *p*-values	–	–	Ins/Del: *p* = 7.8705 $\times 10^{-9}$; dbSNP: *p* = 5.6539 $\times 10^{-9}$

Data are presented as mean ± SD [min–max]. TPs, true positives/retained biological calls in the comparative masking framework; FPs, false positives/artifact-enriched calls excluded by the spatial masking protocol; Ti/Tv, transition/transversion ratio; Ins/Del, insertion/deletion ratio; SD, standard deviation. Statistical significance was evaluated using two-tailed paired *t*-tests comparing retained biological calls with the filtered artifact fraction within each cohort. Raw *p*-values are shown for the mutational-signature and dbSNP-annotation comparisons. *** *p* < 0.0001.
